# Congenital anomalies among live births in a polluted area. A ten-year retrospective study

**DOI:** 10.1186/1471-2393-12-165

**Published:** 2012-12-27

**Authors:** Emilio Antonio Luca Gianicolo, Antonella Bruni, Enrico Rosati, Saverio Sabina, Roberto Guarino, Gabriella Padolecchia, Carlo Leo, Maria Angela Vigotti, Maria Grazia Andreassi, Giuseppe Latini

**Affiliations:** 1 National Research Council, Institute of Clinical Physiology, Campus Ecotekne Building A7, Via per Monteroni, 73100, Lecce, Italy; 2 Division of Neonatology, Perrino Hospital, Brindisi, Italy; 3 National Research Council, Institute of Clinical Physiology, Pisa, Italy; 4 Local Health Unit, Brindisi, Italy; 5 University of Pisa, Pisa, Italy

**Keywords:** Congenital anomalies, Hospital discharge data, Surveillance of birth defects, Registers of congenital anomalies

## Abstract

**Background:**

Congenital anomalies and their primary prevention are a crucial public health issue. This work aimed to estimate the prevalence of congenital anomalies in Brindisi, a city in southeastern Italy at high risk of environmental crisis.

**Methods:**

This research concerned newborns up to 28 days of age, born between 2001 and 2010 to mothers resident in Brindisi and discharged with a diagnosis of congenital anomaly. We classified cases according to the coding system adopted by the European Network for the Surveillance of Congenital Anomalies (EUROCAT). Prevalence rates of congenital anomalies in Brindisi were compared with those reported by EUROCAT. Logistic regression models were adapted to evaluate the association between congenital anomalies and municipality of residence of the mother during pregnancy.

**Results:**

Out of 8,503 newborns we recorded 194 subjects with congenital anomalies (228.2/10,000 total births), 1.2 times higher than the one reported by the EUROCAT pool of registries. We observed 83 subjects with congenital heart diseases with an excess of 49.1%. Odds Ratios for congenital heart diseases significantly increased for newborns to mothers resident in Brindisi (OR 1.75 CI 95% 1.30-2.35).

**Conclusions:**

Our findings indicated an increased prevalence of Congenital Anomalies (especially congenital heart diseases) in the city of Brindisi. More research is needed in order to analyze the role of factors potentially involved in the causation of congenital anomalies.

## Background

Congenital anomalies (CAs) and their primary prevention are an important public health issue and both genetic and environmental factors can contribute to their causation [1]. According to Dolk [2], “Environmental factors include any non-genetic factor that increases the risk of a birth defect for the exposed individual. Such factors are nutritional excesses or deficiencies (e.g. folic acid), maternal illness or infection (e.g. diabetes, rubella), drugs taken during pregnancy (e.g. thalidomide), chemical exposure in the workplace or home (e.g., to solvents or pesticides) and radiation (e.g., medical X-ray).” Scientific literature is interested in the association between CAs and the possible role of chemical contaminants [3], and fetuses are thought to be a further subgroup of the population who could be vulnerable to the effects of air pollutants [4,5].

Brindisi is a town in the Apulia region of southeastern Italy and has a population of about 90,000, covering an area of almost 330 km^2^ with a population density of 271.9 inhabitants per km^2^. Brindisi is the capital of the homonymous province, which has a population of about 402,800 and covers an area of 1,839 km^2^ with a population density of 219.2 inhabitants per km^2^.

**Figure 1:**
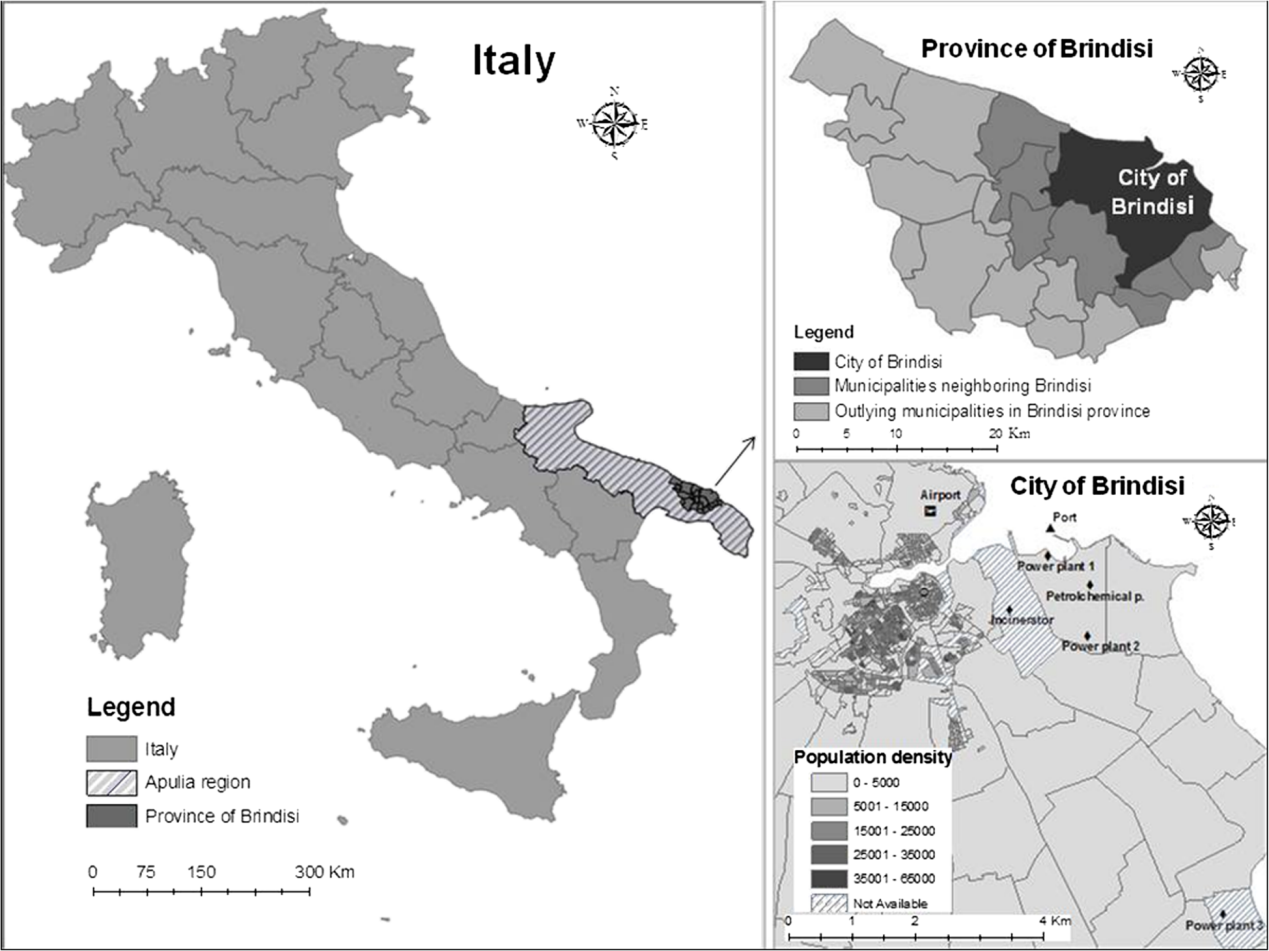
The area of the study: emission sources and population density.

Many sources of pollution are located near the urban area: a huge petrochemical plant, chemical, pharmaceutical, metallurgical and manufacturing plants, an incinerator and three power plants, two of which are coal-fired, producing around 4,400 Megawatts (Figure 1). Moreover, illegal dumps have been found, and the airport and maritime ports, both located in town, have also increased their activity.

In the 1980s, Brindisi and its surrounding municipalities were identified by the Italian Ministry of Environment as an “area at high risk of environmental crisis” [6] and in 1998 the town was included among the 57 sites of the national program of environmental remediation [7]. Epidemiological studies have revealed several critical situations in terms of increasing rates of mortality and morbidity associated with environmental and occupational exposure to pollutants [8-12]. Recently, an analysis of air quality data carried out for the period 1992–2007 revealed the influence of both the industrial area and the harbor on air pollutant concentration in Brindisi [13]. Very recently, although with imprecision due to broad confidence intervals, standardized mortality ratios for congenital anomalies in all ages were observed to be higher than expected (regional reference) in the period 1995–2001 [14]. The authors suggested a plausible role of environmental exposure and they hypothesized the etiological role of pollutants produced both by the petrochemical plant and by dumps [14].

Given this context and in absence of a CA registry, the aim of this study was to estimate the prevalence of congenital anomalies in Brindisi from hospital discharge data (HDD) among newborns in Brindisi between 2001 and 2010 and to compare them with prevalence rates reported by the European network for the surveillance of congenital anomalies (EUROCAT).

## Methods

This study concerned newborns 0–28 days of age, born between 2001 and 2010 to mothers resident in Brindisi and discharged with a diagnosis of CA. Data were extracted from the HDD of the local health unit, which is the regional government unit that holds health records for the entire province of Brindisi, including those related to births. HDD included information on cumulative personal files and healthcare records on diagnoses, the main one and up to five for each discharge record, coded according to the International Classification of Diseases - Clinical Modification, 9th Revision (ICD-9-CM). We considered as cases the newborns with a CA recorded in any of the diagnostic fields. The groups of congenital anomalies analyzed and corresponding ICD-9-CM codes are reported in Table 1.

**Table 1:** **Congenital anomaly subgroups and corresponding ICD**-**9**-**CM codes**

**Congenital anomalies subgroups**	**ICD9**-**CM codes**
Nervous system	740-742
Eye	743
Ear. face and neck	744
Congenital heart disease	745-746, 747–747.4
-*Common arterial truncus*	*745*.*00*
-*Transposition of great vessels*	*745*.*10*
-*Single ventricle*	*745*.*3*
-*Ventricular septal defect*	*745*.*4*
-*Atrial septal defect*	*745*.*5*
-*Atrioventricular septal defect*	*745*.*6*
-*Tetralogy of Fallot*	*745*.*2*
-*Tricuspid atresia and stenosis*	*746*.*1*
-*Ebstein*’*s anomaly*	*746*.*2*
-*Pulmonary valve atresia*	*746*.*01*
-*Pulmonary valve stenosis*	*746*.*02*
-*Aortic valve atresia* / *stenosis*	*746*.*3*
-*Hypoplastic left heart*	*746*.*7*
-*Coarctation of aorta*	*747*.*1*
-*Total anomalous pulm venous return*	*747*.*42*
Respiratory	748
Oro-facial clefts	749.0-749.2
Digestive system	750-751, 756.6
Abdominal wall defects	756.70-756.71, 756.79
Urinary	753, 756.72, 752.62
-*Bladder exstrophy and* / *or epispadia*	*753*.*5*, *752*.*62*
Genital	752.0-752.4, 752.60, 752.61, 752.7-752.9
-*Hypospadia*	*752*.*61*
Limb	754.3-754.8, 755
Musculo-skeletal	754.0-754.2, 756.0-754.5, 756.8-756.9, 762.80
Chromosomal	758.0-758.3, 758.5-758.9
Other malformations	747.5-747.9, 757, 759.0-759.7

We classified cases in the following categories: total CAs, congenital heart disease (CHDs) and other CAs according to the coding system adopted by the European Network for the Surveillance of Congenital Anomalies - EUROCAT. Subcategories of CHDs with at least five cases were also analyzed.

Registries in EUROCAT register information about newborns and the mothers using different data sources [15]. Registries in EUROCAT use the code system of the British Paediatric Association Classification of Diseases ICD-9-BPA, which is a five-digit extension of the ninth revision of the International Classification of Diseases (ICD-9). Thus, in order to have comparable data set we used the corresponding ICD-9-CM codes.

We adopted EUROCAT exclusion criteria for minor anomalies with less medical, functional or cosmetic importance [16]. We excluded cases with patent ductus arteriosus (PDA) in preterm babies (< 37^th^ gestational week) [16]. For this purpose, in order to correctly identify the gestational week (information not recorded on HDD) it was necessary to recover each case-history.

All cases of CA with more than one anomaly were counted only once in the “Total CAs” category.

We calculated observed/expected ratios with 95% confidence interval (CI 95%) [17]: expected cases (E) were obtained by multiplying the proportion of newborns with CAs in the pool of EUROCAT registries [18] by the number of live births to mothers resident in Brindisi.

For each category of CAs, we calculated a rate ratio (RR) obtaining by dividing the prevalence ratios observed among males by the prevalence ratio observed among females [19].

We classified CHDs in the following three classes according to their severity defined on the basis of perinatal mortality rates: Severity I (high perinatal mortality); Severity II (medium perinatal mortality); Severity III (low perinatal mortality) [20]. We also classified CHDs according to the presence or absence of an associated chromosomal anomaly [20].

Together with expert neonatologists and a geneticist, we analyzed 994 records extracted from HDD (11.7% of the total population of newborns) admitted to the local Neonatal Intensive Care Unit (NICU) and registered in an independent and more detailed archive, in order to evaluate the validity of the diagnosis reported in the HDD. Sensitivity, specificity, and positive and negative predictive (PPV and PPN) values were thus calculated in order to estimate validity [21].

For local comparison purposes logistic regression models, adjusted at municipality level for a social economic deprivation index [22] and for maternal average age at delivery, were adapted to evaluate the association between CAs/CHDs and municipality of residence of the mother during pregnancy. For this purpose, municipalities were grouped into three categories: Brindisi, neighboring municipalities and outlying municipalities in Brindisi province.

Statistical analysis was performed using SAS statistical software (SAS Institute Inc, Cary, NC, USA) version 8.2 for Microsoft Windows (Microsoft Corporation, Redmont, WA, USA).

On May 28 2010 (reference number 38131), the ethics committee of the Brindisi local health unit approved our research. Research was in compliance with the Helsinki Declaration. Written informed consent from the parents was obtained.

## Results

Table 2 reports the prevalence rates and the ratio of observed/expected number of anomalies based on the pool of EUROCAT registries. Out of 8,503 live births to mothers resident in Brindisi, 194 newborns showed CAs, 83 a CHD. The prevalence rate of total CAs was 228.2/10,000 newborns. The expected number of cases was 165.5 and the ratio O/E = 117.2 (95% CI 101.8-134.9; p < 0.05) (Table 2). The prevalence rate for the CHDs was 97.6 and the ratio O/E = 149.1 (95% CI 120.2-184.8; p < 0.001). Excesses were observed for each of the subcategories of CHDs considered, with strong evidence against the null hypothesis for Ventricular Septal Defect (179.6; 95% CI 133.7-241.3; p < 0.001) and Pulmonary Valve Stenosis (267.6; 95% CI 133.4-533.2; p < 0.01) (Table 2). Observed cases of Other CAs were close to the expected ones (Table 2).

**Table 2:** Newborns with congenital anomalies born to mothers resident in the city of Brindisi and comparison with the pool of EUROCAT registries

**Subgroups of congenital anomalies**	**Brindisi**		**Pool of EUROCAT registers**				**p**^**a**^
	**Cases observed**	**Rates x 10,000 livebirths**	**Rates x 10,000 livebirths**	**Expected**	**(O/E)x100**	**95% CI**	
All anomalies ^c^	194	228.2	194.6	165.5	117.2	101.8-134.9	*
*Congenital heart disease*	*83*	97.6	65.4	55.7	149.1	*120*.*2*-*184*.*8*	***
- *Ventricular septal defect*	*44*	51.7	28.8	24.5	179.6	133.7-241.3	***
- *Atrial septal defect*	*17*	20.0	19.9	16.9	100.7	**62**.**5**-161.8	
- *Pulmonary valve stenosis*	*8*	9.4	3.5	3.0	267.6	*133*.*4*-*533*.*2*	**
*Other congenital anomalies*	*122*	143.5	153.9	130.9	93.2	**78**.**1**-111.3	

^a^ *P < 0.05; **P < 0.01; ***P < 0.001; ****P < 0.0001.

^b^ Pool of Italian registries.

^c^ Cases of CAs with more than one anomaly were counted only once in “All anomalies” category. Numbers do not add up because of newborns with more than one CA.

Absolute values, rates for 10,000 live births, O/E ratios and CI 95%.

City of Brindisi, 2001–2010.

Out of 83 cases with CHD, we observed 9 cases (10.8%) with medium probability of perinatal mortality (Severity II) and 69 cases (83.1%) with low probability of perinatal mortality (Severity III). No case with high probability of perinatal mortality was registered. Five cases (6%) were not classified because of a poorly specified code. Two cases of CHDs (2.4%) were associated with a chromosomal anomaly.

CAs were more frequent among males (RR = 1.42, 95% CI 1.07-1.89; p < 0.05, not shown in table).

Of 8,503 newborns from mother resident in Brindisi, 994 (11.7%) were admitted to the NICU division and registered in an independent and very detailed archive, which was used for validation purposes. A total of 45 out of 65 met the case definition for one of the major CAs (Table 3) and 24 out of 25 children for CHD (Table 3). Therefore, sensitivity was 69.2% and 96% respectively. The specificity was in both cases almost 100%. PPV was 73.8% for all CAs and 85.7% for CHDs; NPV was 97.9% and 99.9% respectively.

**Table 3:** Validity of hospital discharge data for identifying infants with CAs and CHDs

**Cases identified through Hospital Discharge Data**	**Gold Standard (NICU)**				
	**Yes**	**%**	**No**	**%**	**Total**
ALL CONGENITAL ANOMALIES					
Yes	45	73.8	16	26.2	61
No	20	2.1	913	97.9	933
Total	65	6.5	929	93.5	994
Sensitivity	69.2%		Positive predictive value		73.8%
	95% CI (57.3 - 79.5)			95% CI (61.7 - 83.6)	
Specificity	98.3%		Negative predictive value		97.9%
	95% CI (61.7 - 83.6)			95% CI (96.8 - 98.7)	
CONGENITAL HEART DISEASE					
Yes	24	85.7	4	14.3	28
No	1	0.1	965	99.9	966
Total	25	2.5	969	97.5	994
Sensitivity	96.0%		Positive predictive value		85.7%
	95% CI (81.8 - 99.8)			95% CI (69.1 - 95.3)	
Specificity	99.6%		Negative predictive value		99.9%
	95% CI (99.2 - 99.9)			95% CI (99.5 - 100.0)	

City of Brindisi, at single NICU facility, 2001–2010.

In municipalities neighboring Brindisi 8,669 cases of CAs were observed; 17,523 cases were registered in the outlying municipalities in Brindisi province. The prevalence of CAs is more frequent among newborns to mothers resident in the city of Brindisi (Table 4). Odds Ratios for CHDs were significantly higher in the city of Brindisi (OR = 1.85; 95% CI: 1.36-2.50) then in the outlying municipalities in Brindisi province (Table 5).

**Table 4:** Newborns and cases of CAs according to the residence of the mother during pregnancy

**Residence of the mother during pregnancy**	**All newborns**	**All congenital anomalies**		**Cases with CHDs**^**a**^	
		**Absolute values**	**Cases x 10,000**	**Absolute values**	**Cases x 10,000**
City of Brindisi	8,503	193	227.0	82	96.4
Municipalities neighboring Brindisi	8,669	157	181.1	57	65.8
Outlying municipalities in Brindisi province	17,523	360	205.4	99	56.5

^a^ Cases with diagnosis of PDA were excluded due to the impossibility of identifying the exact gestational week for newborns to mothers resident in municipalities other than Brindisi.

Absolute values and rates for 10,000 live births.

Province of Brindisi, 2001–2010.

**Table 5:** **Crude and adjusted ORs** (**95**% **CI**) **for CAs and CHDs residence of the mother during pregnancy**

**Residence of the mother during pregnancy**	**Unadjusted**			**Adjusted**^**b**^		
	**OR**	**CI 95%**	**P**^**a**^	**OR**	**CI 95%**	**P**^**a**^
ALL CONGENITAL ANOMALIES						
Outlying municipalities in Brindisi province (reference)	1			1		
City of Brindisi	**1**.**13**	**0**.**95**-**1**.**35**		**1**.**13**	**0**.**94**-**1**.**35**	
Municipalities neighbour to Brindisi	**0**.**90**	**0**.**74**-**1**.**09**		**0**.**85**	**0**.**69**-**1**.**05**	
CONGENITAL HEART DISEASE						
Outlying municipalities in Brindisi province (reference)	1			1		
City of Brindisi	**1**.**75**	**1**.**30**-**2**.**35**	***	**1**.**85**	**1**.**36**-**2**.**50**	****
Municipalities neighbour to Brindisi	**1**.**19**	**0**.**86**-**1**.**66**		**1**.**08**	**0**.**75**-**1**.**55**	

^a^ *P < 0.05; **P < 0.01; ***P < 0.001; ****P < 0.0001.

^b^ Models are adjusted at municipality level for a social economic deprivation index and for average maternal age at delivery.

Province of Brindisi, 2001–2010.

## Discussion

Our findings indicated prevalence rate of CAs, and in particular of CHDs, was higher in Brindisi than those reported by the pool of EUROCAT registries and those observed in neighboring municipalities.

For this analysis, 28 days was chosen as maximum age for the diagnosis of CAs; this cut-off might induce a bias comparison since in EUROCAT registries the maximum age for diagnosis has a wide range of variation (1 week to 15 years) [15].

Registries are one of the most accurate approaches in terms of case ascertainment and diagnostic detail, thus assuring reliability in estimating prevalence rates of CAs and a valid base of surveillance systems [23]. Nevertheless, limited resources in birth defect surveillance programs sometimes require the use of electronic health archives -- already available and designed for administrative purposes -- for surveillance by public health researchers [24].

The HDD archive is increasingly used to estimate the occurrence of a wide range of diseases and more recently also to estimate neonatal morbidity and birth defects [25]. HDD were originally designed for administrative purposes, but they can be exploited as an abundant, inexpensive source of epidemiological information on large patient populations [25]. Although issues have been raised regarding geographic variability of archive quality and completeness [26], HDD are considered an important source of information to estimate diseases such as CAs [27].

Together with expert neonatologists and a geneticist, we observed a subset of cases admitted to the NICU and registered in an independent and more detailed archive, in order to evaluate the validity of the diagnosis reported in the HDD. High frequencies of diagnoses correctly recorded was observed. Nevertheless, the validity study was conducted only on a single hospital [21], so the validity of data in other Italian areas must be further demonstrated.

This study could not have identified cases of infants with a CA born at home. Nevertheless, according to the National Institute of Statistics [28], in Italy the percentage of births not occurring in public or private hospitals is 0.1% and it is mainly concentrated in the northern part of Italy. Therefore, this phenomenon can be assumed to be rare in the investigated area and it would not have had any effect on the estimated prevalence of CAs.

Our results on sex differences confirm those reported in literature regarding an increased risk among male newborns [29].

It has recently been suggested that environmental risk factors might play an important role in the etiology of CHD [30-32].

Environmental pollutants of concern for health impact include a wide range of airborne contaminants such as nitrogen dioxide (NO_2_), sulfur dioxide (SO_2_), carbon monoxide (CO), ozone (O_3_); particulate matter (PM) and heavy metals (specifically cadmium, lead, mercury, chromium and arsenic), and organic compounds, specifically dioxins and furans, polychlorinated biphenyls (PCBs), and polycyclic aromatic hydrocarbons (PAHs). Their main sources are traffic, industrial emissions and hazardous waste sites. In recent years, population-based and case–control studies have reported an increased risk of congenital anomalies in polluted areas, especially for selected congenital heart defects [30,33-39].

Some important limitations must be considered when interpreting the results of this study.

First, environmental data at the individual or ecological level were not available.

Secondly, we were not able to adjust our analysis for potential confounding factors that are known or suspected to increase the risk of congenital anomalies, such as maternal diabetes, smoking, parental occupation and social determinants, because they were unavailable in the HDD archives [40,41].

Thirdly, data covered live births only. In estimating the prevalence of CAs in a population, stillbirths and pregnancy terminations should be taken into account. Many fetuses die in utero and technological advances in diagnosis provide a growing possibility of reaching termination of pregnancy. This is a very important issue that needs to be addressed in estimating prevalence of CAs but could not be tackled in the present study. However, although the frequency of voluntary abortion in Italy in the years 2006–2007 (230 x 1,000 newborns) was lower than the European one (244) [42], in Apulia the frequency was much higher (304) than in the other Italian regions [43]. Thus, the higher CA rates observed in Brindisi might not be biased by pregnancy terminations. However, this aspect together with the study of incidents of CAs among stillbirths should be further investigated.

## Conclusions

Our data showed the validity of HDD in estimating the prevalence of CAs in the city of Brindisi. Furthermore, an increased prevalence of CAs (especially CHDs) in the city of Brindisi, known to be an area at high risk of environmental crisis, was observed. More research in terms of etiological studies is needed, in order to analyze the role of different risk factors in the possible causation of CAs. In this work we did not have any personal or ecological exposure data. Therefore, it was impossible to infer about the possible role of pollution. Nevertheless, our working group is now focusing on exposure data and on the correlation between environmental data and health data. This will be the aim of our next paper.

Results from this kind of study ought to reduce this burden through identifying the best strategies for primary prevention.

## Abbreviations

CAs: Congenital Anomalies; HDD: Hospital Discharge Data; EUROCAT: European Surveillance of Congenital Anomalies; ICD-9-CM: International Classification of Diseases - Clinical Modification, 9^th^ Revision; CHDs: Congenital Heart Diseases; PDA: Patent Ductus Arteriosus; NICU: Neonatal Intensive Care Unit; PPV: Positive Predictive Value; PPN: Negative Predictive Value; CI: Confidence Interval; E: Expected; O: Observed; RR: Rate Ratio; NO_2_: Nitrogen Dioxide; SO_2_: Sulfur Dioxide; CO: Carbon Monoxide; O_3_: Ozone; PM: Particulate Matter; PCBs: Polychlorinated Biphenyls; PAHs: Polycyclic Aromatic Hydrocarbons.

## Competing interests

The authors declare no competing interests.

## Authors’ contributions

EALG, analyzed data, reviewed the literature and was the main contributor to writing the manuscript. AB, managed the newborns data, the record linkage and contributed to writing the manuscript. ER, contributed in analysing each case-history and validating the diagnosis of congenital anomalies. SS and RG were responsible for data recovery from NICU data set and contributed to writing the manuscript. GP and LC provided Hospital Discharge Data. MAV and MGA reviewed the literature and the final manuscript. GL, was Senior Physician and contributed to writing the manuscript. All authors read and approved the final manuscript.

## Pre-publication history

The pre-publication history for this paper can be accessed here:

http://www.biomedcentral.com/1471-2393/12/165/prepub
